# Complete Genome Sequence of *Mycoplasma synoviae* Strain WVU 1853^T^

**DOI:** 10.1128/genomeA.00563-15

**Published:** 2015-05-28

**Authors:** Meghan A. May, Gerald F. Kutish, Anthony F. Barbet, Dina L. Michaels, Daniel R. Brown

**Affiliations:** a Department of Biomedical Sciences, College of Osteopathic Medicine, University of New England, Biddeford, Maine, USA; b Department of Pathobiology and Veterinary Science and Center of Excellence for Vaccine Research, University of Connecticut, Storrs, Connecticut, USA; c Department of Infectious Diseases and Pathology, College of Veterinary Medicine, University of Florida, Gainesville, Florida, USA

## Abstract

A hybrid sequence assembly of the complete *Mycoplasma synoviae* type strain WVU 1853^T^ genome was compared to that of strain MS53. The findings support prior conclusions about *M. synoviae*, based on the genome of that otherwise uncharacterized field strain, and provide the first evidence of epigenetic modifications in *M. synoviae*.

## GENOME ANNOUNCEMENT

The genome of *Mycoplasma synoviae* type strain WVU 1853^T^ has presented an assembly challenge, especially because of the many contiguous pseudogenes encoding alleles of its primary cytadhesin VlhA (1, 2). This most important virulence factor of *M. synoviae* is not represented in the genome survey of strain WVU 1853^T^ (GenBank GCA_000385095.1). Illumina HiSeq paired-end (600× coverage) and PacBio single-molecule real-time (200× coverage) sequencing of DNA we extracted from first-passage specimens of ATCC 25204, lot 59130888, were performed by the Scripps Research Institute Sequencing Core and the University of Florida Interdisciplinary Center for Biotechnology Research, respectively. Hybrid assembly was achieved in January 2015 by using a combination of Celera (3), Newbler (4), Ray (5), Sprai (http://zombie.cb.k.u-tokyo.ac.jp/sprai/index.html), and *proovread* (6) software, and then annotated via NCBI’s Prokaryotic Genome Annotation Pipeline (7). Nucleotide and amino acid sequence similarities to the genome of *M. synoviae* field strain MS53 (8) were calculated using JSpecies version 1.2.1 (9) and AAI Calculator (http://enve-omics.ce.gatech.edu/aai). Epigenetic modifications were analyzed using the PacBio RS_Modification_and_Motif_Analysis module version 2.2.0.

The closed circular genome is 846,495 bp in length, with 97% average nucleotide and 99% predicted amino acid identity to strain MS53. Its G+C content is 28.3 mol% versus the 34.2% estimated by buoyant density (10). The tetranucleotide frequency correlation with strain MS53 is 0.9985. Differences from strain MS53 include the presence of genes encoding type I restriction, modification, and specificity system subunits, and greater numbers of mobile element and hypothetical proteins. A single-nucleotide deletion in its clustered regularly interspaced short palindromic repeat (CRISPR)-associated *csn1* open reading frame (ORF) VY93_03200 likely accounts for the absence of a CRISPR array from this lineage of WVU 1853^T^; extensive strain-specific arrays occur in strain MS53 (8) and in *M. synoviae* strains K3344 and K5016 (our unpublished data). Its 78.5-kb *vlhA* locus, consisting of approximately 60 promoterless pseudogenes of varying lengths, is 33.7 mol% G+C and 14% longer than that of MS53. The expressed *vlhA* allele VY_01465 has at best 86% nucleotide similarity to others reported for different lineages of strain WVU 1853^T^ but does encode the sialoreceptor binding motif PKVTFNLAAKEG (11). Modified bases consistent with methylation by N-6 adenine-specific DNA methylases clustered into four common (2,146 to 3,840 instances) and eight less common (7 to 313 instances) motifs distributed throughout the genome (modification quality scores, all > 30; *P* < 0.001). Between 84 and 88% of all instances of the most common motifs were methylated. Only 18 to 45% of all instances of six less common motifs were methylated, while the two least common motifs (7 and 37 instances) were 100% and 92% methylated, respectively. Additional m6A-modified bases and 742 putative instances of methylation by N-4 cytosine-specific DNA methylase (*P* < 0.001) were not clustered. Candidate DNA methylases involved included the predicted Dam methylases VY93_01015 and VY93_02585, Dcm methylase VY93_02380, the type I methylation subunit VY93_03755, and type III system subunits VY93_00870 and VY93_03705. These findings support prior conclusions about *M. synoviae* based on the genome of the otherwise uncharacterized strain MS53 and provide the first evidence of epigenetic modifications of the *M. synoviae* genome.

### Nucleotide sequence accession numbers.

The *M. synoviae* WVU 1853^T^ genome sequence and annotation data have been deposited in GenBank under the accession number CP011096; the sequence described in this paper is the first version, CP011096.1.
